# Impact of Personal Cooling on Performance, Comfort and Heat Strain of Healthcare Workers in PPE, a Study From West Africa

**DOI:** 10.3389/fpubh.2021.712481

**Published:** 2021-09-01

**Authors:** Ana Bonell, Behzad Nadjm, Tida Samateh, Jainaba Badjie, Robyn Perry-Thomas, Karen Forrest, Andrew M. Prentice, Neil S. Maxwell

**Affiliations:** ^1^Medical Research Council Unit The Gambia at London School of Hygiene and Tropical Medicine, Banjul, Gambia; ^2^Centre on Climate Change and Planetary Health, London School of Hygiene and Tropical Medicine, London, United Kingdom; ^3^Environmental Extremes Laboratory, University of Brighton, Eastbourne, United Kingdom

**Keywords:** personal protective equipment, heat strain, cooling, cognitive function, healthcare workers, occupational heat strain

## Abstract

**Background:** Personal protective equipment (PPE) is an essential component of safely treating suspected or confirmed SARS-CoV-2 patients. PPE acts as a barrier to heat loss, therefore increasing the risk of thermal strain which may impact on cognitive function. Healthcare workers (HCWs) need to be able to prioritize and execute complex tasks effectively to ensure patient safety. This study evaluated pre-cooling and per-cooling methods on thermal strain, thermal comfort and cognitive function during simulated emergency management of an acutely unwell patient.

**Methods:** This randomized controlled crossover trial was run at the Clinical Services Department of the Medical Research Unit The Gambia. Each participant attended two sessions (Cool and Control) in standard PPE. Cool involved pre-cooling with an ice slurry ingestion and per-cooling by wearing an ice-vest external to PPE.

**Results:** Twelve participants completed both sessions. There was a significant increase in tympanic temperature in Control sessions at both 1 and 2 h in PPE (*p* = 0.01). No significant increase was seen during Cool. Effect estimate of Cool was −0.2°C (95% CI −0.43; 0.01, *p* = 0.06) post 1 h and −0.28°C (95% CI −0.57; 0.02, *p* = 0.06) post 2 h on tympanic temperature. Cool improved thermal comfort (*p* < 0.001), thermal sensation (*p* < 0.001), and thirst (*p* = 0.04). No difference on cognitive function was demonstrated using multilevel modeling.

**Discussion:** Thermal strain in HCWs wearing PPE can be safely reduced using pre- and per-cooling methods external to PPE.

## Introduction

SARS-CoV-2 continues to cause significant mortality and morbidity worldwide (1). Healthcare systems and healthcare workers (HCW) must ensure effective and timely treatment of cases without compromising safety (2). For HCWs this involves use of personal protective equipment (PPE) when treating suspected or confirmed cases, following international guidelines (3, 4).

PPE is a physical barrier preventing viral contamination, however it also reduces evaporative and radiative heat loss leading to potential uncompensable heat load, thermal strain and discomfort (5). Acknowledging this, the Center for Disease Control and Prevention (CDC), the American Conference of Governmental Industrial Hygienists (ACGIH) and the International Organization for Standardization (ISO) have all produced guidelines to ensure workers' safety in thermal extremes (6–8). However even if these guidelines are known about and adhered to, recent studies have questioned whether the measures are sufficient in tropical climates (9, 10).

In tropical regions there are several factors that increase the risk of thermal strain. Ambient environmental conditions are likely to be high. For example in The Gambia, West Africa, average daily temperatures range from 29 to 34°C with annual average levels of relative humidity at 68%, significantly higher than recommended temperature and humidity for indoor surgical operating theaters (25°C and 60%) (11). Additionally, in most healthcare facilities in resource-limited tropical settings, natural ventilation systems alone are often relied upon, with limited availability of air-conditioning (12). Wall-mounted air-conditioning units, where available, recirculate air without a HEPA filter and are advised against by the WHO (13). Ceiling or standing fans are not recommended in any but single occupancy rooms. Therefore, there is a high environmental heat load that is difficult to mitigate. Concerns regarding shortages of PPE mean that healthcare workers often wear PPE for prolonged periods. The length of time in PPE increases the risk of dehydration, thermal strain, physical exhaustion and may compromise decision making (14–16). During this current pandemic, many HCW are wearing PPE for 4 or more hours (17).

The most appropriate personal cooling mechanism for healthcare workers in PPE likely differs depending on work load, environmental stressors and resources (5). Comparative evidence from industry and athletes have shown internal, external and mixed-method cooling to reduce thermal strain (18–25).

Pre-cooling (reducing body temperature prior to heat exposure) with ice slurry ingestion (ISE) lowers core temperature and increases heat storage capacity, delaying the onset of sweating and risk of dehydration, reducing thermal discomfort and improving endurance capabilities (26, 27). ISE is more effective than water ingestion at absorbing heat and can therefore have a greater impact on reducing body temperature (28). It also improves perception of effort, cognitive function and fatigue (29). However, the effects of ISE are time-limited (30). Ice-vests have been shown to improve endurance performance and thermal comfort via changes in skin temperature, although they do not lower core temperature as ISE does (18, 21). Mixed methods of cooling, including pre and per (during)-cooling methods have been found to be the most effective (18). Studies of ice-vests with PPE to date have placed the ice-vests under PPE (18, 21), however after several hours the ice packs will melt and will then add to physical discomfort and energy cost. A simple effective cooling mechanism for HCW in PPE has not been established.

Despite conflicting evidence of the impact of heat strain on simple mental tasks, there is growing consensus that above 38.2°C core temperature, dual-task performance and complex-task sharing are negatively impacted by heat strain (31–34). This is particularly relevant to HCWs who are often caring for multiple patients, need to be able to prioritize tasks effectively and perform accurate calculations, all under stress. There is little literature on the impact of heat stress on HCWs' ability to perform routine tasks in tropical conditions (11). Studies from temperature-controlled settings give conflicting evidence of the effect of PPE on HCW emergency tasks performance, where clinical tasks performed by specialists appear to be preserved from the impact of the physical effects of PPE (i.e., anesthetists vs. clinicians on intubation) (35, 36). One study on surgeons' ability to perform laparoscopic operative tasks at 26 vs. 19°C found a significant increase in both physical demand and distractibility at higher temperatures (37). Another study evaluating different PPE suits at 22 and 28°C did not show any impact on simulated HCW tasks (38).

We hypothesize that PPE-induced thermal strain impairs complex task performance by HCWs and this effect will be mitigated by personal cooling methods. This study aimed to be directly transferable to clinical practice and therefore was simple and pragmatic, assessed the risk of compromising PPE and assessed the ability to perform life-saving procedures. We evaluated the use of a combination of pre-cooling with ISE ingestion and per-cooling via ice-vests external to PPE on thermal strain, thermal comfort and cognitive function during simulated emergency management of an acutely unwell patient.

## Materials and Methods

### Participants

We enrolled 16 HCWs from the pool of staff working on the COVID wards in the Clinical Services Department of the Medical Research Unit The Gambia (MRCG). We used convenience sampling of non-pregnant, non-shielding staff. Written informed consent was given by all participants. Baseline characteristics are presented in Table 1. All participants were long-term residents of The Gambia. All experiments described below took place around the usual staff shifts.

**Table 1:** Mean (SD) demographic and anthropometric measurements of participants.

	**Males**	**Females**	***p*-value**
N (%)	7 (58%)	5 (42%)	
Ethnicity: Gambian	6	2	
Other West African country	1	3	
Occupation: Qualified nurse	4	4	
Auxiliary nurse (HCA)	3	1	
Chronic medical conditions	0	0	
Age (years) Mean (SD)	29.4 (2.6)	36.5 (12.2)	0.28
Weight (kg) Mean (SD)	68.0 (15.6)	80.2 (24.4)	0.31
Height (cm) Mean (SD)	184.2 (11.6)	171.4 (7.5)	0.06
BMI^*^ (kg/m^2^) Mean (SD)	19.8 (2.67)	26.9 (6.38)	**0.02**

* *Body mass index. Bold indicates p value < 0.05*.

Ethics approval was granted by the Gambia Government/MRC joint ethics committee and the London School of Hygiene and Tropical Medicine Ethics Advisory Board (Ref. 22590). The study was conducted in accordance with the Institution's ethics and governance committee, and Declaration of Helsinki (39).

### Experimental Design

This was a randomized controlled crossover, repeated measures experiment of ISE and ice-vests (Cool) on thermal strain, thermal comfort, and cognitive function in HCW in PPE. Each participant was invited to attend two sessions (Cool vs. Control) at least 4 days apart to minimize any further acclimation effect from repeated heat exposures, at the same time of day to avoid the effect of diurnal rhythms on core temperature.

### Sample Size

Sample size calculation was based on results from Quinn et al.'s study of cooling methods in PPE in environmental conditions designed to reproduce conditions in West Africa (40). In this study core temperature in control (38.86C) and ice-vest intervention (37.94C) gave an effect size of 0.92. Taking an alpha of 0.05 and a power of 0.8, a minimum sample size requirement of 12 would detect a similar difference.

### Session Protocol and Simulation Training

Sessions occurred in an unoccupied hospital ward. Environmental conditions were measured using the HT200: Heat Stress WBGT (Wet Bulb Globe Temperature) Meter (Extech^®^, NH, USA). The first hour mimicked a teaching ward round covering the WHO Basic Emergency Care course and the Advanced Life Support Algorithm from the UK Resuscitation Council (41, 42). This hour was spent standing or sitting taking notes with an estimated metabolic equivalent task (MET) of 1.8. There followed a revision quiz and simulation training. During the simulation all participants had to deliver effective cardiopulmonary resuscitation (CPR) and bag-mask ventilation to a mannequin. The estimated METs of CPR were 5.7 (43). Sessions were delivered by an Advanced Life Support Instructor and medical doctor and were tailored to locally available equipment. Sessions lasted ~2 h, with some extension to allow completion of the cognitive function tests.

### PPE

All participants wore standard PPE for treating suspected or confirmed covid-19 patients, as specified by MRCG@LSHTM. This consisted of scrubs under category III type 5B/6B protection coveralls with hood, shoe covers, gloves, an FFP2 mask, and face shield.

### Measurements

Tympanic temperature measurements were taken using a Braun ThermoScan^®^ 7 tympanic thermometer (Braun GmbH, Kronberg, Germany). Duplicate measurements were taken from both left and right tympanic. The highest measurement was taken from the four readings at each time point (44–46).

Heart rate and blood pressure were measured whilst sitting from the right arm using an OMRON M3 automatic device (Omron, Kyoto, Japan). These were measured hourly throughout the sessions.

Urine specific gravity was measured by urine dipstick and urine osmolality with a portable refractometer, calibrated daily (Osmocheck™, TECIL, Barcelona, Spain).

Thermal comfort was measured on a six-point scale from very comfortable (1) to very uncomfortable (6). Thermal sensation was measured on an eight-point scale from very cold (1) to unbearably hot (8). Thirst was measured on a five-point scale from not thirsty (1) to very, very thirsty (5).

### Cognitive Function Test

A cognitive battery test was used to assess overall cognitive function. CogniFit General Cognitive Assesment^®^ is a computer-based series of exercises which test multiple cognitive domains and is widely used (47, 48)^1^. The program gives an overall score and specific scores for 26 cognitive areas. All participants completed a familiarization visit with the program prior to attending the study sessions.

### Cooling Intervention

**Figure 1:**
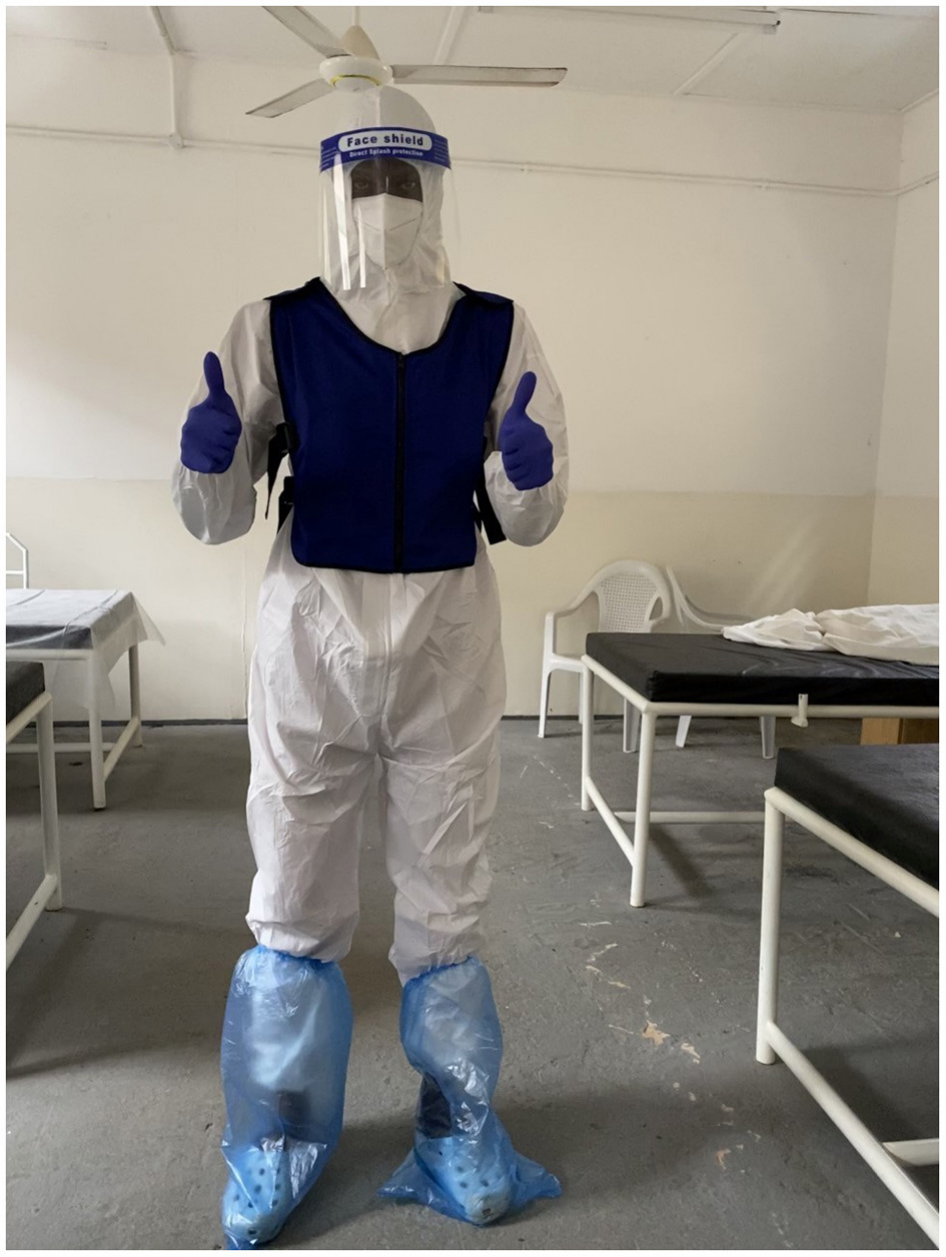
A participant in full PPE with ice vest.

For the Cool session, participants were given 7.5 ml/kg of ice slurry to drink over 15 min immediately prior to donning PPE (26). Once full PPE was applied, a commercially available outdoor cooling vest to protect against heat stroke was put on (Sports Cooling Vest, Desertcart.com^©^). The cooling vest was a sleeveless, zipped vest with 6 pockets for ice packs, two at the front and four at the back. Ice packs were placed in the vest at the start of the session and replaced hourly (Figure 1).

### Statistical Analyses

Data are reported as mean ± SD, all continuous variables were assessed for normality by distribution and Shapiro-Wilk normality test. Baseline physiology and environmental conditions between control and intervention sessions were assessed using paired *t*-test for normally distributed variables and Wilcox Signed-Rank test for non-parametric variables.

One-way ANOVA was used to determine the change in temperature or heart rate over time in Cool and Control. However, due to violation of model assumptions for repeated measures ANOVA, a multilevel model was used to assess change in tympanic temperature (model 1), heart rate (model 1), and cognitive function (model 2) with intervention as a fixed effect and individuals as random effects. The overall cognitive score and four pre-determined outputs from the cognitive battery test: divided attention, focused attention, and shifting and working memory, were analyzed (model 2). These four outputs were chosen to correlate with the need to multi-task whilst remaining focused as a healthcare worker.

Model 1:

$$\begin{array}{l} Y\;\sim \;\beta_{0}+\beta_{1}*intervention+\;\left(1|ind\right) \end{array}$$

Model 2:

$$\begin{array}{l} Cognitive\;score\;\sim \;\beta_{0}+\beta_{1}*intervention+\beta_{2}*session+\beta_{3}*order\\ \;+\beta_{4}*sex+\beta_{5}*age+\beta_{6}*T_{\text{tymp}}+\;\left(1|ind\right)\\ \;Session=session\;1\;or\;2\\ \;Order=order\;of\;intervention\;by\;session\\ \;Sex=male\;or\;female\\ \;Age=age\;of\;participant\;in\;years\;at\;recruitment\\ \;T_{\text{tymp}}=maximum\;tympanic\;temperature\\ \;\left(1|ind\right)=participant\;as\;a\;random\;effect \end{array}$$

The difference between thermal comfort, sensation and thirst for control and intervention at the end of the session were assessed by proportions and chi-squared. Thermal comfort was redefined as comfortable (very comfortable to just comfortable) and uncomfortable (just uncomfortable to very uncomfortable). Thermal sensation was redefined as not hot (very cold to warm) and hot (hot to unbearably hot). Thirst was redefined as minimal thirst (not thirsty to slightly thirsty) and thirsty (thirsty to very, very thirsty).

## Results

Sixteen participants were recruited and participated in the first session. Three participants (2 male and 1 female) were unable to attend the second session. One participant (female) was acutely unwell during session two, was subsequently diagnosed with an acute viral infection and removed from the study.

Twelve registered and auxiliary nurses, 7 males and 5 females, completed both Control and Cool sessions. Of these, one participant terminated the simulation early during the Control session due to light-headedness and pre-syncope. All participants completed the Cool session.

Mean air temperature, relative humidity and wet bulb globe temperature (WBGT) at baseline were 29.3°C, 69.3% and 26.1°C, respectively. Average temperature, humidity and WBGT throughout the sessions were 30.2°C (range 28.8–32.0°C), 68.2% (range 53.8–75.3%), and 27.2°C (range 25.0–29.3°C), respectively. There was no statistically significant difference in climate exposure between Cool and Control sessions (*p* = 0.42).

### Baseline

Baseline physiology of participants at the start of each session were similar: heart rate (Control = 79.1; Cool = 80.8 bpm), *t*_(11)_ = −0.2, *p* = 0.82 and urine osmolality (Control = 695; Cool = 628 mOsm/kg), *t*_(11)_ = 0.6, *p* = 0.54 and Wilcox Signed-Rank test for tympanic temperature (Control = 36.8; Cool 37.0°C), *Z* = 10.5, *p* = 0.17 and urine specific gravity (Control = 1.03; Cool = 1.03), *Z* = 16, *p* = 0.79.

### Physiology

**Figure 2:**
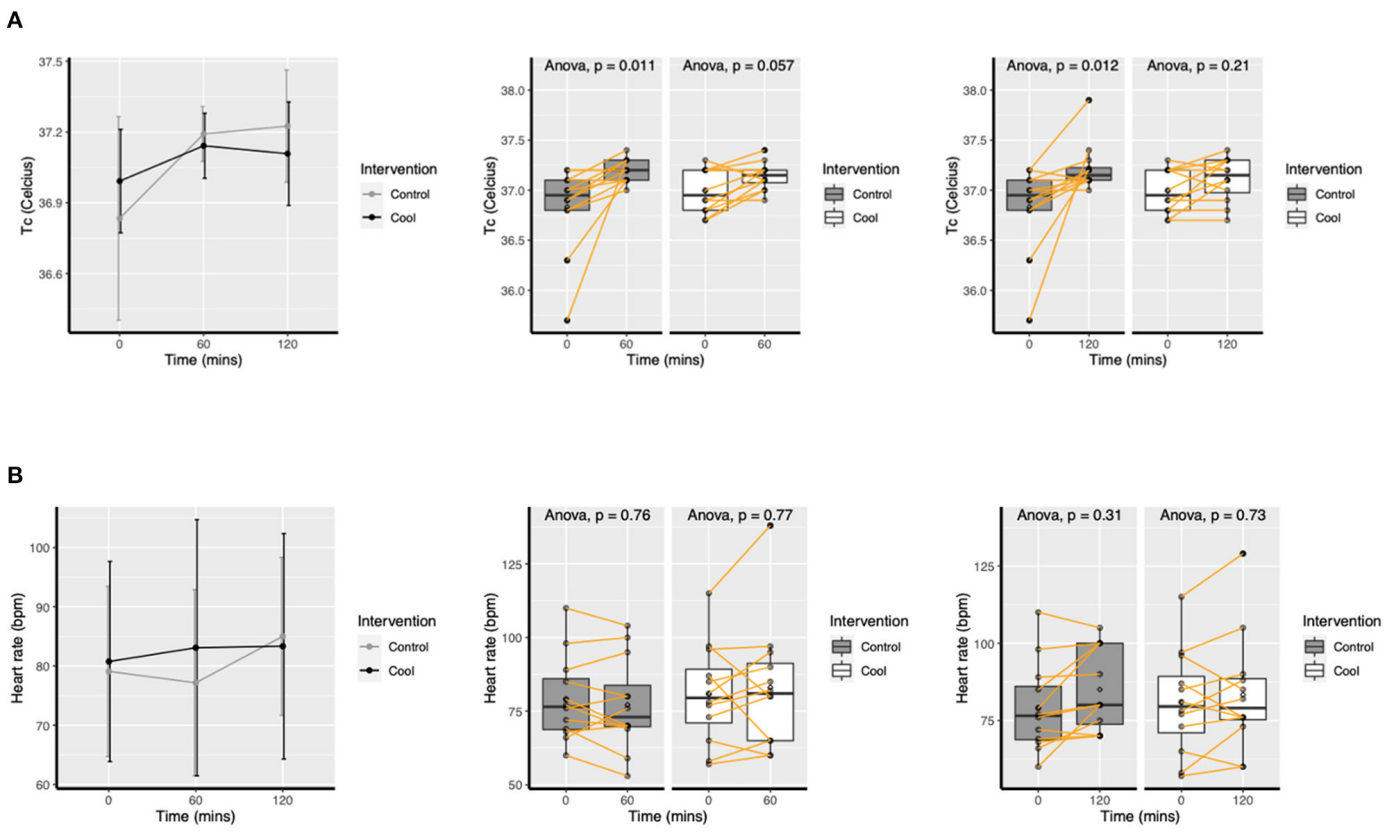
Mean (SD) change in tympanic temperature **(A)** and heart rate **(B)** over time for Cool and Control.

Mean tympanic temperature change are presented in Figure 2. There was a significant increase in tympanic temperature in the Control group from baseline to 1 h in PPE (*p* = 0.01) and 2 h in PPE (*p* = 0.01), but no significant increase in Cool (*p* = 0.06, *p* = 0.21 at hour 1 & 2, respectively). Heart rate did not change in either Control or Cool sessions (see supplement).

Multilevel modeling gave an effect estimate of Cool as −0.2°C, 95% CI −0.43; 0.01, *p* = 0.06 post 1 h and as −0.28°C, 95% CI −0.57; 0.02, *p* = 0.06 post 2 h on tympanic temperature.

### Perception

**Figure 3:**
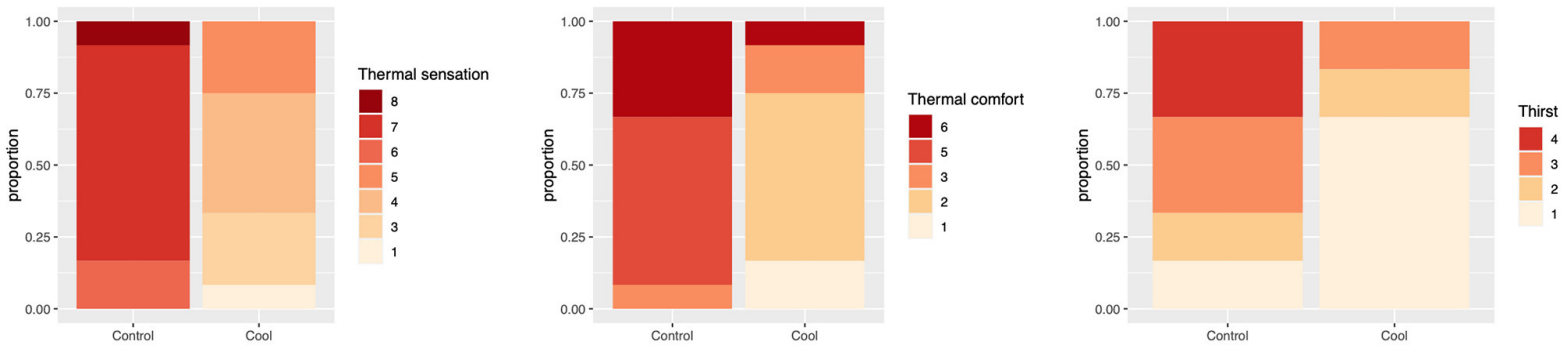
Thermal sensation, comfort and thirst by Control and Cool.

Thermal sensation, comfort and thirst all differed significantly between control and intervention (Figure 3). For thermal comfort 92% (11/12) were uncomfortable in the control vs. 8% (1/12) in the intervention (*p* < 0.001). For thermal sensation 100% (12/12) of those in the control arm felt hot or above at the end of the session vs. 0% (0/12) in the intervention arm (*p* < 0.001). For thirst, 83% (10/12) felt thirsty or very thirsty in the control vs. 17% (2/12) in the intervention (*p* = 0.04).

### Cognitive Function

Mean cognitive function scores per session are presented in Table 2.

**Table 2:** Mean (SD) Cognitive function results by order of test performed and Control vs. Cool.

**Cognitive function test**	**Familiarization test**	**Session 1 test**	**Session 2 test**
Mean (SD)	150 (66.5)	208 (87.1)	224 (101.9)
Range	46–273	106–366	79–380
	**Control**	**Cool**	
Mean (SD)	222 (102.5)	210 (86.8)	
Range	94–380	79–366	

The first test was the familiarization test and each subsequent test was run after completion of the sessions whilst participants remained in PPE. The standard error of measurement (SEM) indicated there was a learning element to the test where the test completed at the end of session 2 scored higher than the session 1 test (familiarization: session 1 SEM = 19.0%, session 1: session 2 SEM = 17.2%.

Multilevel modeling did not demonstrate any difference in overall cognitive function by Control vs. Cool. Table 3 gives details of the models for all pre-specified cognitive domains tested. Intervention had no impact on any domains.

**Table 3:** Linear multilevel model estimates for different cognitive parameters.

	**Estimate [95% CI]**	***P*-value for LRT**	**Estimate [95% CI]**	***P*-value for LRT**
	*Total cognitive function score*		*Divided attention*	
Session 1	63.2 [24.6, 102.6]	** <0.001**	103.6 [−48.1, 255.1]	0.43
Session 2	76.2 [42.7, 109.1]		19.3 [−110.3, 149.0]	
Order	54.8 [−25.2,134.7]	0.24	193.0 [57.6, 328.7]	**0.01**
Intervention	−7.6 [−40.5, 25.6]	0.67	−83.6 [−212.8, 54.5]	0.25
Tc	1.3 [−130.5, 146.1]	0.99	458.8 [−9.7, 923.6]	0.08
Age	−4.2 [−9.2, 0.7]	0.14	10.3 [2.0, 18.7]	**0.03**
Sex	24.1 [−44.4, 92.4]	0.54	−23.1 [−142.0, 95.6]	0.73
	*Focused attention*		*Shifting*	
Session 1	60.5 [−73.7, 192.7]	0.40	76.3 [−81.9, 228.2]	0.41
Session 2	82.4 [−31.0, 196.8]		92.7 [−38.0, 226.6]	
Order	46.1 [−87.5, 179.9]	0.56	247.9 [59.3, 437.1]	**0.02**
Intervention	13.6 [−100.1, 126.7]	0.82	40.2 [−92.4, 170.9]	0.57
Tc	−37.0 [−463.9, 368.5]	0.87	534.3 [−22.7, 1015.6]	0.06
Age	−5.3 [−13.6, 2.9]	0.27	6.5 [−5.1, 18.2]	0.34
Sex	−40.4 [−156.6, 76.0]	0.55	107.1 [−55.8, 270.9]	0.26
	*Working memory*			
Session 1	79.9 [17.4, 144.2]	** <0.001**		
Session 2	135.9 [81.5, 189.4]			
Order	18.3 [−120.6, 157.0]	0.82		
Intervention	−15.0 [−68.4, 38.9]	0.60		
Tc	−193.5 [−407.3, 47.3]	0.11		
Age	−5.7 [−14.3, 2.9]	0.25		
Sex	−31.1 [−150.0, 87.4]	0.65		

*Bold indicates p value < 0.05*.

### Model Validity

Plots of all multilevel model residuals were examined for deviation from linear form by Pearson correlation. Constance of variance of residuals was also examined and normality of residuals. All model assumptions in all models were met according to these tests.

## Discussion

This study demonstrated that a combination of pre- and per-cooling reduced thermal strain in HCW wearing PPE, and can improve discomfort from thermal sensation, comfort and thirst. We did not show any impact on cognitive function.

Several recent studies have explored personal cooling options whilst wearing PPE and although our findings are similar to these, this study is novel in that it evaluated a mixed-method cooling approach (using both internal and external, and pre and per-cooling) and the effect of external cooling was prolonged by replacing ice-packs. The study by De Korte et al. explored 21°C phase change vests under PPE and significantly improved thermal comfort and sensation, although it had no impact on thermal strain (49). However, the transferability of this vest for cooling in the tropics is questionable due to differences in ambient conditions and potential resource limitation. Another study of PPE used for Ebola treatment, based in a heat-chamber, found a reduction in heat strain with several different types of cooling vest (40). Although this study was performed in similar ambient conditions, the PPE requirements for Ebola are different to COVID and the exposure was only for 1 h and so not directly transferrable to current practice in treating COVID.

Focusing on the physiological changes in the study, we did not see the rise in temperatures described in two other studies of PPE use in West Africa (50, 51). These studies were performed during the Ebola outbreak and found HCW in PPE had an average core temperature rise to 38°C and in 4/25 individuals exceeded 38.5°C after 1 h in PPE (50). This may in part be due to the characteristics of the PPE requirements for Ebola vs. COVID, since external conditions were similar, but may in part be due to heat acclimation. Our study's participants were West African nationals, who had been residing in the region for at least 6 months prior to the study and therefore had likely acclimated (a series of phenotypic changes resulting in physiological alterations that act to protect against heat stress) and the other studies participants were French and American nationals. Notably, mean resting tympanic temperature was lower in the participants of the present study.

This may also help to explain why there was no impact of wearing PPE on cognitive function amongst HCW. The literature on the impact of heat stress on cognitive function suggests a critical threshold of thermal strain on cognitive function, below which there is no impact (31). The HCW in our study did not cross this critical threshold, potentially explaining the lack of impact on cognitive function. This is reassuring for patient care, although our data was measured on a single day and it is unclear whether cumulative thermal strain over successive days wearing PPE would result in a diminished cognitive function.

All participants in the study were able to perform emergency medical procedures with no evidence of compromise to the PPE, condensation or droplet spread caused by the per-cooling vest. The practicalities of enacting this intervention will depend on the facilities available. In the MRCG hospital, we have completed staff sensitization and awareness sessions, provided access to cold/iced drinks in the hospital staff common room and located a chest freezer, the ice-packs and ice-vests in the COVID-zone. This will enable staff to use the ice-vests in a contaminated space and keep them in that space, avoiding the risk and inconvenience of repeated decontamination.

There are several limitations to the study. There were four participants who were unable to complete both sessions, reducing the sample size. The sample size calculation was based on previous studies of heat alleviation in PPE, and so although we did not meet the target of sixteen, twelve was within our minimal sample required. At baseline, the tympanic temperature of the Cool group was 0.2C higher than the Control and although this is likely due to chance and was not a statistically significant difference, it may have impacted on the findings. Additionally, although the temperature and humidity remained relatively constant during the session, it did vary more than if the study had been run in a heat-chamber, but there was no significant difference between Cool and Control sessions. The advantage of the study being run on a hospital ward was that although the ambient conditions were not controlled, they were exactly what HCWs experience and so directly generalizable. The gold standard for core temperature is rectal or esophageal temperature. These were not practical whilst in PPE and cost constraints prevented the use of a telemetry pill. Core temperature may thus have under-recorded, however we monitored both the change in temperature as well as the absolute temperature to reduce the impact of this limitation on data interpretation. Additionally, most physiological studies use continuous heart rate monitoring, which was not available in this study. The cognitive battery test used was designed and validated in America and assumes a degree of computer or tablet literacy resulting in certain language and technological aptitude barriers. Finally, the study used simulation to model real-life ward experience, to ensure standardization, but in reality, clinical work can be highly variable, and this may not have been captured. Additionally, shift durations vary dramatically depending on staffing, clinical workload and availability of PPE and therefore the applicability of these solutions will be locally dependent. However, direct comparison of Control and Cool would have been very challenging in a real-world setting.

## Conclusion

Pre- and per- cooling using internal and external cooling modalities reduced thermal strain in HCW in PPE for a prolonged duration, dramatically improved thermal sensation, comfort and thirst and is safe to implement, with no detriment on ability to perform medical tasks or contamination risk by condensation.

## Data Availability Statement

The raw data supporting the conclusions of this article will be made available by the authors, without undue reservation.

## Ethics Statement

The studies involving human participants were reviewed and approved by Gambia Government/MRC joint ethics committee and the London School of Hygiene and Tropical Medicine Ethics Advisory Board. The patients/participants provided their written informed consent to participate in this study. Written informed consent was obtained from the individual(s) for the publication of any potentially identifiable images or data included in this article.

## Author Contributions

AB conceived, designed, implemented, and completed first draft of the manuscript. BN advised on design, facilitated implementation, and edited manuscript. TS and JB facilitated implementation and edited manuscript. RP-T, KF, and AP advised on design and edited manuscript. NM advised on conception and design and edited manuscript. All authors contributed to the article and approved the submitted version.

## Conflict of Interest

The authors declare that the research was conducted in the absence of any commercial or financial relationships that could be construed as a potential conflict of interest.

## Publisher's Note

All claims expressed in this article are solely those of the authors and do not necessarily represent those of their affiliated organizations, or those of the publisher, the editors and the reviewers. Any product that may be evaluated in this article, or claim that may be made by its manufacturer, is not guaranteed or endorsed by the publisher.

## Footnotes

^1^Available online at: https://www.cognifit.com/cognitive-research-tool.

## References

[1] WHO. COVID-19 Weekly Epidemiological Update. World Health Organization (2021). Available online at: https://www.who.int/publications/m/item/weekly-epidemiological-update—5-january-2021

[2] Ha JF. The COVID-19 pandemic, personal protective equipment and respirator: a narrative review. Int J Clin Pract. (2020) 74:e13578. 10.1111/ijcp.13578 32511834 PMC7300506

[3] Ortega R Gonzalez M Nozari A Canelli R. Personal protective equipment and COVID-19. N Engl J Med. (2020) 382:e105. 10.1056/NEJMvcm2014809 32427435

[4] WHO. Rational Use of Personal Protective Equipement for Coronavirus Disear (COVID-19) and Considerations During Severe Shortages. World Health Organization (2020). Contract No.: WHO/2019-nCoV/IPC_PPE_use/2020.4.

[5] Foster J Hodder SG Goodwin J Havenith G. Occupational heat stress and practical cooling solutions for healthcare and industry workers during the COVID-19 pandemic. Ann Work Expo Health. (2020) 64:915–22. 10.1093/annweh/wxaa082 32955080 PMC7543286

[6] ACGIH. Appendix B. ACGIH Threshold Limit Values (TLVs) and Biological Expsoure Indices (BEIs). 1330 Kemper Meadow Drive, Cincinnati, OH, USA: ACGIH (2012).

[7] ISO. ISO 7933:2004(E). ISO (2004).

[8] Jacklistsch BW John Williams W Musolin K Coca A Kim J-H Turner N. NIOSH Criteria for a Recommended Standard: Occupational Exposure to Heat and Hot Environments. U.S. Department of Health and Human Services, Centres for Disease Control and Prevention, National Institute for Occupational Safety and Health: NIOSH (2016). Contract No.: 2016-106.

[9] Meade RD Poirier MP Flouris AD Hardcastle SG Kenny GP. Do the threshold limit values for work in hot conditions adequetaley protect workers? Med Sci Sports Exerc. (2016) 48:1187–96. 10.1249/MSS.0000000000000886 26938043

[10] Meshi EB Kishinhi SS Mamuya SH Rusibamayila MG. Thermal exposure and heat illness symptoms among workers in mara gold mine, Tanzania. Ann Glob Health. (2018) 84:360–8. 10.29024/aogh.2318 30835389 PMC6748306

[11] Byrne J Ludington-Hoe SM Voss JG. Occupational heat stress, thermal comfort, and cognitive performance in the OR: an integrative review. Aorn J. (2020) 111:536–45. 10.1002/aorn.13009 32343372

[12] Yau YH Chandrasegaran D Badarudin A. The ventilation of multiple-bed hospital wards in the tropics: a review. Build Environ. (2011) 46:1125–32. 10.1016/j.buildenv.2010.11.013 32288016 PMC7116949

[13] WHO. Module 1B: Ventilation and Exhausted Air Treatment as IPC Measures Within a COVID-19 Context. World Health Organisation (2020).

[14] Maynard SL Kao R Craig DG. Impact of personal protective equipment on clinical output and perceived exertion. J R Army Med Corps. (2016) 162:180–3. 10.1136/jramc-2015-000541 26511850

[15] Williams JC Krah Cichowicz J Hornbeck A Pollard H Snyder J. NIOSH Science Blog [Internet]. Prevention CfDCa, editor (2020). Available online at: https://blogs.cdc.gov/niosh-science-blog/2020/06/10/ppe-burden/ (accessed January 5, 2021).

[16] AlGhamri AA Murray SL Samaranayake VA. The effects of wearing respirators on human fine motor, visual, and cognitive performance. Ergonomics. (2013) 56:791–802. 10.1080/00140139.2013.767383 23514088

[17] Tabah A Ramanan M Laupland KB Buetti N Cortegiani A Mellinghoff J. Personal protective equipment and intensive care unit healthcare worker safety in the COVID-19 era (PPE-SAFE): an international survey. J Crit Care. (2020) 59:70–5. 10.1016/j.jcrc.2020.06.005 32570052 PMC7293450

[18] Bach AJE Maley MJ Minett GM Zietek SA Stewart KL Stewart IB. An evaluation of personal cooling systems for reducing thermal strain whilst working in chemical/biological protective clothing. Front Physiol. (2019) 10:424. 10.3389/fphys.2019.00424 31031643 PMC6474400

[19] Bongers CCWG Hopman MTE Eijsvogels TMH. Cooling interventions for athletes: an overview of effectiveness, physiological mechanisms, and practical considerations. Temperature. (2017) 4:60–78. 10.1080/23328940.2016.1277003 28349095 PMC5356217

[20] Selkirk GA McLellan TM Wong J. Active versus passive cooling during work in warm environments while wearing firefighting protective clothing. J Occup Environ Hyg. (2004) 1:521–31. 10.1080/15459620490475216 15238305

[21] Kenny GP Schissler AR Stapleton J Piamonte M Binder K Lynn A. Ice cooling vest on tolerance for exercise under uncompensable heat stress. J Occup Environ Hyg. (2011) 8:484–91. 10.1080/15459624.2011.596043 21756138

[22] Caldwell JN Patterson MJ Taylor NA. Exertional thermal strain, protective clothing and auxiliary cooling in dry heat: evidence for physiological but not cognitive impairment. Eur J Appl Physiol. (2012) 112:3597–606. 10.1007/s00421-012-2340-x 22328005

[23] Glitz KJ Seibel U Rohde U Gorges W Witzki A Piekarski C. Reducing heat stress under thermal insulation in protective clothing: microclimate cooling by a “physiological” method. Ergonomics. (2015) 58:1461–9. 10.1080/00140139.2015.1013574 25679096

[24] Ioannou LG Tsoutsoubi L Mantzios K Gkikas G Piil JF Dinas PC. The impacts of sun exposure on worker physiology and cognition: multi-country evidence and interventions. Int J Environ Res Public Health. (2021) 18:7698. 10.3390/ijerph18147698 34300148 PMC8303297

[25] Ioannou LG Mantzios K Tsoutsoubi L Nintou E Vliora M Gkiata P. Occupational heat stress: multi-country observations and interventions. Int J Environ Res Public Health. (2021) 18:6303. 10.3390/ijerph18126303 34200783 PMC8296111

[26] Siegel R Maté J Brearley MB Watson G Nosaka K Laursen PB. Ice slurry ingestion increases core temperature capacity and running time in the heat. Med Sci Sports Exerc. (2010) 42:717–25. 10.1249/MSS.0b013e3181bf257a 19952832

[27] Stanley J Leveritt M Peake JM. Thermoregulatory responses to ice-slush beverage ingestion and exercise in the heat. Eur J Appl Physiol. (2010) 110:1163–73. 10.1007/s00421-010-1607-3 20714767

[28] Douzi W Dugué B Vinches L Al Sayed C Hallé S Bosquet L. Cooling during exercise enhances performances, but the cooled body areas matter: a systematic review with meta-analyses. Scand J Med Sci Sports. (2019) 29:1660–76. 10.1111/sms.13521 31340407

[29] Saldaris JM Landers GJ Lay BS. Enhanced decision making and working memory during exercise in the heat with crushed ice ingestion. Int J Sports Physiol Perform. (2019). 10.1123/ijspp.2019-0234. [Epub ahead of print]. 31711038

[30] Bolster DR Trappe SW Short KR Scheffield-Moore M Parcell AC Schulze KM. Effects of precooling on thermoregulation during subsequent exercise. Med Sci Sports Exerc. (1999) 31:251–7. 10.1097/00005768-199902000-00008 10063814

[31] Bruyn LL Lamoureux T. Literature review: cognitive effects of thermal strain. Defence Techn Inform Center. (2005) 35.

[32] Schmit C Hausswirth C Le Meur Y Duffield R. Cognitive functioning and heat strain: performance responses and protective strategies. Sports Med. (2017) 47:1289–302. 10.1007/s40279-016-0657-z 27988874

[33] Vasmatzidis I Schlegel RE Hancock PA. An investigation of heat stress effects on time-sharing performance. Ergonomics. (2002) 45:218–39. 10.1080/00140130210121941 11964205

[34] Ramsey JD. Task performance in heat: a review. Ergonomics. (1995) 38:154–65. 7875117 10.1080/00140139508925092

[35] Schumacher J Arlidge J Dudley D Sicinski M Ahmad I. The impact of respiratory protective equipment on difficult airway management: a randomised, crossover, simulation study. Anaesthesia. (2020) 75:1301–6. 10.1111/anae.15102 32335900 PMC7267320

[36] Schumacher J Gray SA Michel S Alcock R Brinker A. Respiratory protection during simulated emergency pediatric life support: a randomized, controlled, crossover study. Prehosp Disaster Med. (2013) 28:33–8. 10.1017/S1049023X12001525 23089080

[37] Berg RJ Inaba K Sullivan M Okoye O Siboni S Minneti M. The impact of heat stress on operative performance and cognitive function during simulated laparoscopic operative tasks. Surgery. (2015) 157:87–95. 10.1016/j.surg.2014.06.012 25482467

[38] Loibner M Hagauer S Schwantzer G Berghold A Zatloukal K. Limiting factors for wearing personal protective equipment (PPE) in a health care environment evaluated in a randomised study. PLoS ONE. (2019) 14:e0210775. 10.1371/journal.pone.0210775 30668567 PMC6342303

[39] World Medical Association. World Medical Association Declaration of Helsinki: ethical principles for medical research involving human subjects. JAMA. (2013) 310:2191–4. 10.1001/jama.2013.281053 24141714

[40] Quinn T Kim JH Strauch A Wu T Powell J Roberge R. Physiological evaluation of cooling devices in conjunction with personal protective ensembles recommended for use in West Africa. Disaster Med Public Health Prep. (2017) 11:573–9. 10.1017/dmp.2016.209 28303772 PMC9903158

[41] WHO-ICRC. Basic Emergency Care: Approach to the Acutely Ill and Injured. ABCDE and SAMPLE History Approach. WHO (2018).

[42] UK RC. Advanced Adult Life Support: Teaching Material. UK RC (2015).

[43] Valdez JE Edson S Emmel M Rivera-Macias J Adams B Casilla M. Energy expenditure performing hands-only cardiopulmonary resuscitation during average emergency response times. In: EWU Digital Commons. Cheney: Eastern Washington University (2018).

[44] Brinnel H Cabanac M. Tympanic temperature is a core temperature in humans. J Ther Biol. (1989) 14:47–53. 10.1016/0306-4565(89)90029-6

[45] Sato KT Kane NL Soos G Gisolfi CV Kondo N Sato K. Reexamination of tympanic membrane temperature as a core temperature. J Appl Physiol. (1985). (1996) 80:1233–9. 10.1152/jappl.1996.80.4.1233 8926251

[46] Yeoh WK Lee JKW Lim HY Gan CW Liang W Tan KK. Re-visiting the tympanic membrane vicinity as core body temperature measurement site. PLoS ONE. (2017) 12:e0174120. 10.1371/journal.pone.0174120 28414722 PMC5393563

[47] Peretz C Korczyn AD Shatil E Aharonson V Birnboim S Giladi N. Computer-based, personalized cognitive training versus classical computer games: a randomized double-blind prospective trial of cognitive stimulation. Neuroepidemiology. (2011) 36:91–9. 10.1159/000323950 21311196

[48] Haimov I Hanuka E Horowitz Y. Chronic insomnia and cognitive functioning among older adults. Behav Sleep Med. (2008) 6:32–54. 10.1080/15402000701796080 18412036

[49] de Korte JQ Bongers CCWG Catoire M Kingma BRM Eijsvogels TMH. Cooling vests alleviate perceptual heat strain perceived by COVID-19 nurses. Temperature. (2020):1–11. 10.1080/23328940.2020.1868386 PMC9154750 35655667

[50] Grélot L Koulibaly F Maugey N Janvier F Foissaud V Aletti M. Moderate thermal strain in healthcare workers wearing personal protective equipment during treatment and care activities in the context of the 2014 Ebola virus disease outbreak. J Infect Dis. (2016) 213:1462–5. 10.1093/infdis/jiv585 26655297

[51] Coca A Quinn T Kim JH Wu T Powell J Roberge R. Physiological evaluation of personal protective ensembles recommended for use in West Africa. Disaster Med Public Health Prep. (2017) 11:580–6. 10.1017/dmp.2017.13 28303774 PMC9901493

